# Nurses' attitudes toward the use of robots in healthcare

**DOI:** 10.1002/nop2.1435

**Published:** 2022-11-23

**Authors:** Makoto Yamanaka, Kohei Kajiwara, Jun Kako, Takuya Yasumoto

**Affiliations:** ^1^ Japanese Aichi Medical Unversity School of Nursing Ngakute‐City Japan; ^2^ Japanese Red Cross Kyushu International College of Nursing Munakata Japan; ^3^ College of Nursing Art and Science University of Hyogo Akashi Japan; ^4^ School of Nursing Sugiyama Jogakuen University Nagoya Japan

**Keywords:** attributes, nursing, robots

We read the article by Zrinyi et al. (2022), titled “Nurse preferences of caring robots: A conjoint experiment to explore most valued robot features,” which was published in the *Nursing Open* on 27 June 2022. Zrinyi et al. (2022) assessed nurses' potential perceptions of robots by examining previous studies and utilizing symbolic words used by nurses in reference to robots as responses that depict their impressions.

The authors reported that the survey population included approximately 13% of nurses who had worked with robots before. We believe that considering whether nurses possess such experience is necessary to assess their perceptions of robots accurately.

Yen et al. (2018) surveyed nursing tasks and reported that nurses spend approximately 10% of their time on replaceable tasks that do not need to be performed by nurses. Additionally, they reported that the quality of nursing tasks is improved when performed by non‐nurses (Yen et al., 2018). Robots can perform tasks that do not need to be performed by nurses and are deployed to perform some of these tasks in order to reduce nurses' workload. However, Robert (2019) reported that while robots can perform about 60% of nursing tasks, they take 20 times longer.

We therefore believe that nurses who have worked with robots before may have held expectations of a reduced workload but witnessed robots' slow performance of tasks and are thus significantly biased in their perception of working with robots. Zrinyi et al. (2022) also stated that “Due to the very low number of participants exposed to nursing robots, we were not able to conduct a separate analysis to support previous findings. Therefore, we could not confirm the positive views of nurses who had already worked with caring robots” (p. 4). Therefore, we believe that it is necessary only to analyse nurses who have not worked with robots before to increase the reliability of the results of this study.

Second, we believe that the authors' findings with regard to nurses' concerns about robots taking on a role related to the core value of nursing care are interesting. Reports have indicated that companion robots have had a positive psychosocial impact on patients. However, we believe that the nurses' perception of robots as an unfavourable substitute for communication and the relatively low perceived safety of operation have important implications for the widespread use of robots in the medical field. Yen et al. (2018) reported in their survey that nurses spent much of their time communicating with patients in their rooms as part of their duties.

The results indicated that a fair share of physicians and other healthcare professionals recognize the importance of gathering information through communication with patients and that robots cannot replace this (Roter & Hall, 1995). In addition, the authors believe that the relatively low importance of operational safety is largely due to the fact that robots have been proven to be safe in medical settings and because companion robots are beginning to become more prevalent in daily life. We believe that this result's value in the nursing field would be enhanced by incorporating the nurses’ identities into the authors' discussion. The consolidation of the authors' argument through this addition will increase their credibility and consequently promote the diffusion of robots, which are sure to enter the medical field.
